# Differential Uptake of Antisense Oligonucleotides in Mouse Hepatocytes and Macrophages Revealed by Simultaneous Two-Photon Excited Fluorescence and Coherent Raman Imaging

**DOI:** 10.1089/nat.2021.0059

**Published:** 2022-06-01

**Authors:** Prabuddha Mukherjee, Edita Aksamitiene, Aneesh Alex, Jindou Shi, Kajari Bera, Chi Zhang, Darold R. Spillman, Marina Marjanovic, Michael Fazio, Punit P. Seth, Kendall Frazier, Steve R. Hood, Stephen A. Boppart

**Affiliations:** ^1^ GSK Center for Optical Molecular Imaging and University of Illinois at Urbana-Champaign, Urbana, Illinois, USA.; ^2^ Beckman Institute for Advanced Science and Technology, University of Illinois at Urbana-Champaign, Urbana, Illinois, USA.; ^3^ In vitro/In vivo Translation, Research, GlaxoSmithKline, Collegeville, Pennsylvania, USA.; ^4^ Department of Electrical and Computer Engineering and University of Illinois at Urbana-Champaign, Urbana, Illinois, USA.; ^5^ Department of Bioengineering, University of Illinois at Urbana-Champaign, Urbana, Illinois, USA.; ^6^ Carle Illinois College of Medicine, University of Illinois at Urbana-Champaign, Urbana, Illinois, USA.; ^7^ Ionis Pharmaceuticals, Inc., Carlsbad, California, USA.; ^8^ In vitro/In vivo Translation, Research, GlaxoSmithKline, Stevenage, United Kingdom.

**Keywords:** PS-ASO, multimodal imaging, GalNAc, hepatocytes, macrophages

## Abstract

Antisense oligonucleotides (ASOs), a novel paradigm in modern therapeutics, modulate cellular gene expression by binding to complementary messenger RNA (mRNA) sequences. While advances in ASO medicinal chemistry have greatly improved the efficiency of cellular uptake, selective uptake by specific cell types has been difficult to achieve. For more efficient and selective uptake, ASOs are often conjugated with molecules with high binding affinity for transmembrane receptors. Triantennary N-acetyl-galactosamine conjugated phosphorothioate ASOs (GalNAc-PS-ASOs) were developed to enhance targeted ASO delivery into liver through the hepatocyte-specific asialoglycoprotein receptor (ASGR). We assessed the kinetics of uptake and subsequent intracellular distribution of AlexaFluor 488 (AF488)-labeled PS-ASOs and GalNAc-PS-ASOs in J774A.1 mouse macrophages and primary mouse or rat hepatocytes using simultaneous coherent anti-Stokes Raman scattering (CARS) and two-photon fluorescence (2PF) imaging. The CARS modality captured the dynamic lipid distributions and overall morphology of the cells; two-photon fluorescence (2PF) measured the time- and dose-dependent localization of ASOs delivered by a modified treatment of suspension cells. Our results show that in macrophages, the uptake rate of PS-ASOs did not significantly differ from that of GalNAc-PS-ASOs. However, in hepatocytes, GalNAc-PS-ASOs exhibited a peripheral uptake distribution compared to a polar uptake distribution observed in macrophages. The peripheral distribution correlated with a significantly larger amount of internalized GalNAc-PS-ASOs compared to the PS-ASOs. This work demonstrates the relevance of multimodal imaging for elucidating the uptake mechanism, accumulation, and fate of different ASOs in liver cells that can be used further in complex *in vitro* models and liver tissues to evaluate ASO distribution and activity.

## Introduction

Antisense oligonucleotides (ASOs) represent a new paradigm and drug discovery platform, different from traditional small molecule and protein therapeutics [1]. Gapmer ASOs bind complementary RNA in cells by Watson–Crick base pairing and promote its degradation by recruiting Ribonuclease H1 that selectively cleaves the RNA strand of a DNA/RNA heteroduplex [2]. While the primary advantage of ASOs is the specificity by which they can downregulate target RNA, the major challenge is their delivery to selective cellular targets [3]. Specifically, targeted delivery of ASOs to liver hepatocytes is attractive as it provides an opportunity to treat liver diseases, including Hepatitis B, a double-stranded DNA virus that affects millions of people in the world [4].

Triantennary N-acetyl-galactosamine (GalNAc) terminated oligosaccharides show high affinity for the transmembrane asialoglycoprotein receptor (ASGR), a C-type Lectin that is highly and exclusively expressed in liver hepatocytes [5–11]. The ASGR is constitutively internalized into hepatocytes by clathrin-mediated endocytosis [8]. After internalization, the ASGR-GalNAc-PS-ASO complex is trafficked into endo-lysosomal compartments where it dissociates in the acidic environment to liberate the receptor which is recycled back to the plasma membrane.

The liver is composed of hepatocytes that make up 80% of the liver mass and nonparenchymal cells, including endothelial, Kupffer, and stellate cells, that make up 10% of the organ mass. Unconjugated PS-ASOs accumulate preferentially in nonparenchymal cells of the liver mediated by the protein-binding properties of the PS backbone [12]. The PS backbone enhances protein binding and drives cellular uptake of ASOs in the absence of targeting ligands [13]. GalNAc conjugation reverses this trend and promotes ASO uptake into hepatocytes that results in ∼10-fold improvement in potency for gene targets expressed in hepatocytes [7]. As a result of this preclinical animal model study, ASO gapmers (with and without GalNAc) were developed to target a region shared among all Hepatitis B virus (HBV) mRNAs [14,15].

While the preliminary study results using transgenic mice and healthy human volunteers suggested a more pronounced accumulation of GalNAc-PS-ASOs in the liver [15], this difference was less apparent in patients with HBV infection [16]. This observation raised the question whether ASGR distribution or function in virally infected hepatocytes is altered or whether the internalization of GalNAc-ASOs is impaired. Thus, elucidation of mechanisms of the clinical efficacy of gapmer ASOs in Hepatitis B patients is required.

To investigate the efficacy of PS-ASO delivery in HBV-infected liver cells, we adopted a stepwise approach. Before working with intricate three-dimensional tissue/organoid models and two-dimensional cocultures, we sought to evaluate ASO internalization in isolated hepatic cell cultures. Our study was conducted on primary rodent hepatocytes and immortalized mouse monocyte/macrophage-like J774A.1 cells as these are the cell types that are relevant to HBV infection and PS-ASO uptake, respectively, in the liver [17].

Similar to primary peritoneal macrophages, J774A.1 cells express dendritic cell markers CD86 and CD11c and macrophage mannose receptors and galactose-type lectin (MGL) receptors, a homolog of ASGR [18,19]. We utilized AlexaFluor488 (AF488) dye-labeled ASOs targeting mouse scavenger receptor B (SRB1) mRNA for our studies as the unconjugated and GalNAc conjugated versions of these PS-ASOs previously demonstrated improved potency in mice as a result of improved distribution to hepatocytes [7].

Fluorescence imaging has been used consistently for detecting PS-ASO uptake, endosomal release, and intracellular trafficking in mammalian cells [20]. In all these studies, however, the PS-ASOs were tagged with a fluorescent label, and the conclusions regarding PS-ASO internalization were derived from measuring the dynamics of these labels. A direct visualization of the PS-ASO internalization inside living cells is still lacking.

Vibrational imaging presents us with the opportunity to encounter this challenge due to its unique advantages of spatial–chemical specificity to small molecules, subcellular resolution, and minimal physiological perturbation [21]. Coherent anti-Stokes Raman scattering (CARS) microscopy is one such label-free, high-resolution, vibrational imaging technique that can acquire spatiotemporal and compositional information from biological systems within minutes [22–24] and has been used extensively to probe lipid structures [25–30] in a biochemical environment.

In our laboratory, CARS microscopy is available in two varieties: (a) hyperspectral, where we sweep a vibrational frequency range, and (b) single frequency, where we target one specific vibrational mode. This second form of CARS microscopy is highly compatible with any other optical imaging modality and can be measured simultaneously. Since the PS-ASOs were already tagged with the AF488 dye, we measured simultaneous single frequency CARS and fluorescence images to characterize the PS-ASO uptake processes and their trafficking through the fluorescence images and, at the same time, assess the changes in the cellular morphology and the corresponding cellular lipid distribution from CARS.

Findings from our work highlight key uptake and trafficking differences for GalNAc conjugated and unconjugated PS-ASOs. This work also demonstrates the utility of multimodal imaging for elucidating ASO uptake pathways in different cell types in the liver and the potential utility of this technology for understanding uptake pathways for other classes of oligonucleotide therapeutics.

## Materials and Methods

### ASO synthesis

The PS-ASOs and the GalNAc-PS-ASOs reported here were designed against the SRB1 sequence as previously reported [7]. AF488 dye label was conjugated to these ASOs as follows. Parent SRB1 ASO was prepared using standard phosphoramidite chemistry on an AKTA 10 synthesizer. Parent compound with 3′ GalNac and 5′ amine was purified using SAX HPLC chromatography. Finally, AF488 was conjugated using standard NHS-amine coupling chemistry in 1:1 0.1 M Borate buffer, pH 8.5: DMF at 100 mg/mL concentration. The final compound was purified using SAX HPLC chromatography, followed by C18 desalt and evaporation.

### Cell cultures and treatment with ASO

Adherent immortalized mouse ascite-derived J774A.1 (ATCC^®^ TIB-67™) monocyte/macrophage cells were routinely maintained in a complete phenol-free high D-glucose (25 mM) and HEPES (pH 7.4) containing Dulbecco's modified Eagle's medium (DMEM) (Thermo Fisher Scientific; Cat #21063029) and supplemented with 10% HyClone characterized heat-inactivated fetal bovine serum (FBS) (Cytiva Life Sciences), 4 mM L-Glutamine, and 1% penicillin–streptomycin–amphotericin B solution in a humidified 5% CO_2_ incubator at 37°C.

For imaging studies, 12.5 mL of fresh media was added to T-75 flasks with cells; then cells were dislodged with a cell scraper, aspirated, and counted to determine total and viable cell number by a Trypan Blue dye exclusion assay. The main reason we did not use trypsin is because such treatment may greatly influence the cell membrane protein composition, reactivity (at least transiently) of the cell to medium components, cell membrane permeability, and even differentiation/phenotype state—variables that would impact our comparative ASO uptake study, especially knowing that the corresponding hepatocytes have not undergone enzymatic treatment.

Therefore, in our study, the mechanical stress evoked by harvesting the attached subpopulation of cells was chosen over the conditions that would introduce any unwanted variables like integrity of the cell surface reporters and allocating sufficient time for them to recover. To remove aggregates, cell suspensions were mixed with phenol-free TrypLE™ Select Enzyme (1X) cell dissociation reagent (Thermo Fisher Scientific) at a 1:1 ratio and then analyzed using an automated Beckman Coulter Vi-CELL XR cell counter and cell viability analyzer with default mammalian cell type settings. Relative cell viability percentage (CV%) was calculated using the following formula:

(1) $$Cellviability\left(\%\right)=\frac{Totalnumberofviablecells}{Totalnumberofcells}\times 100.$$

For reverse transfection with indicated ASOs, a 0.5 × 10^6^ cell/mL suspension with a mean CV% of 85% was dispensed into 15 mL Eppendorf DNA LoBind conical tubes that contained the stock solution of ASOs and was further diluted to the indicated final concentrations (ranging from 50 nM to 5 μM). The vial with control cells contained the corresponding volume of PBS. The tubes were centrifuged at 200 *g* for 5 min at room temperature, the supernatant was mixed with the cell pellet, and 2.5 mL of reverse transfected cells were seeded into sterile poly-D-Lysine-coated 35 mm imaging dishes with #0 14 mm glass coverslips (MatTek Life Sciences). The cells were measured immediately or at indicated time intervals. Maximum cell incubation time with ASOs was 24 h.

Cryopreserved CD-1 primary mouse hepatocytes (PMHs) were purchased from Thermo Fisher Scientific (Cat #MSCS10, Lots #MC829 and #MC881) and thawed as recommended by the manufacturer using a prewarmed thawing medium (phenol-free Williams Medium E supplemented with Hepatocyte Plating Supplement Pack). Thereafter, thawed hepatocytes were resuspended in prewarmed hepatocyte incubation medium (phenol-free Williams Medium E supplemented with Hepatocyte Maintenance Supplement Pack, containing dexamethasone and a cocktail solution of penicillin–streptomycin), ITS+ (insulin, transferrin, selenium complex, bovine serum albumin, and linoleic acid), GlutaMAX™, and HEPES (Thermo Fisher Scientific).

CV% and yield were determined by manual Trypan Blue dye exclusion assay. Mean Lot #MC829 hepatocyte post-thaw viability was 78.8% (compared to 89% reported by the supplier). Mean Lot #MC881 hepatocyte post-thaw viability was 88% (compared to 87% reported by the supplier). After 1.5 and 3 h of being maintained in a suspension on a 2D tube revolving rotator, mean hepatocyte viability declined to 71% and 62.14%, respectively.

Cryopreserved primary hepatocytes isolated from Sprague-Dawley male rats were purchased from Thermo Fisher Scientific (# RTCS10, Lot #RS978) and thawed as described above. Average post-thaw viability of cells was 80% (compared to 86% reported by the supplier). After 1.5 h of incubation in a suspension in a humidified 5% CO_2_ incubator at 37°C, cell viability was 60%. A representative cell viability measurement for primary rat hepatocytes is shown in Supplementary Fig. S1, after 60%.

For reverse transfection with indicated ASOs, a 0.25 × 10^6^ cells/mL primary cell suspension was dispensed into 15 mL Eppendorf DNA LoBind conical tubes that contained the stock solution of ASOs and was further diluted to the indicated final concentrations. The vial with control cells contained the corresponding volume of PBS. The tubes were centrifuged at 55 *g* for 1 min at RT, the supernatant was mixed with the cell pellet, and 2.5 mL of reverse transfected cells were seeded into sterile poly-D-Lysine-coated 35 mm imaging dishes with #0 14 mm glass coverslips (MatTek Life Sciences). The cells were allowed to settle in a humidified 5% CO_2_ incubator at 37°C before imaging at designated time intervals. Maximum cell incubation time with ASOs was 3 h. All measurements were performed in technical and biological triplicates.

### Fluorescence microscopy

After 24 h (for J774A.1 cells) or 1.5 h (for hepatocytes) post-treatment with ASOs, a separate set of plates was used to evaluate cell viability by total and dead cell staining with cell-permeable Hoechst 33342 and cell-impermeable propidium iodide (PI) dyes. Fluorescence in DAPI (blue) and TexasRed (red) channels was measured using a 10 × /0.50 FLUAR objective in a Zeiss AXIO Observer inverted microscope (Carl Zeiss Microscopy LLC, White Plains, NY). ASO fluorescence was detected using the FITC (green) channel.

### Multimodal optical imaging

**FIG. 1.**
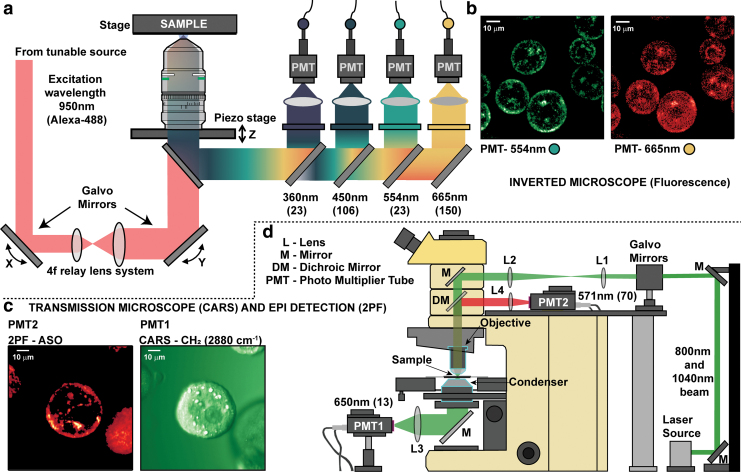
Optical imaging microscopes used in this study. **(a)** The inverted microscope arrangement where a 950 nm femtosecond pulse is used to excite the AlexaFluor 488 (AF488)-tagged PS-ASOs to evaluate their cellular uptake patterns. **(b)** A representative image of PMH from the AF488 fluorescence from the 550 nm channel (*green*) and overall cellular morphology from the 665 nm channel (*red*). **(c)** Simultaneous CARS (*green*) and 2PF (*red*) images of mouse primary hepatocytes obtained from the transmission microscope. **(d)** The layout of the transmission microscope that uses the transmission geometry to acquire the CARS images and the reflection geometry to acquire the 2PF images. 2PF, two-photon fluorescence; CARS, coherent anti-Stokes Raman scattering; PMH, primary mouse hepatocyte. Color images are available online.

The details of the multimodal imaging setup were described previously [31,32]. Briefly, the system was composed of two individual microscopes as shown in Fig. 1, one that can acquire multiphoton excited fluorescence and detect multiharmonic generation from the sample, and the other being capable of acquiring CARS and 2PF simultaneously. These two microscopes will be further referred to in the text as the “inverted” (Fig. 1a, b) and “transmission” (Fig. 1c, d) microscopes, respectively.

### Two-PF imaging

We used a dual output, broadly tunable 80 MHz femtosecond laser source (Chameleon Discovery, Coherent, Inc.) to generate the laser excitation pulses for both microscopes. The tunable output was centered at 950 nm so that the two-photon excitation at 475 nm predominantly excites the AF488 dye (Ex/Em = 490/525 nm). The laser beam was coupled into a custom-built inverted microscope designed for live cell/tissue imaging applications. A 25X, 1.05 NA objective (Olympus XLPLN25XWMP2) was used for focusing the laser beam onto the cells, and the fluorescence signal was collected in epi-fluorescence (epi) mode.

Our microscope was designed to collect optical signals from various nonlinear processes from 340 nm to 700 nm in four different channels for select spectral ranges as shown in Fig. 1a, of which we utilized the third (green) and fourth (yellow) channels for this study. We collected the 2PF of AF488 by integrating the emission signals from 542 nm to 566 nm using a bandpass filter (FF-01-554/23–25, Semrock), and the emission was detected using a photon counting photo-multiplier tube (PMT; Hamamatsu H7421-40). The fourth channel was primarily used to detect autofluorescence signal from the endogenous cofactor flavin adenine di-nucleotide (FAD).

The 2PF emission signals of FAD ranging from 593 nm to 700 nm were filtered using a bandpass filter (FF-01-665/150–25; Semrock) and subsequently detected using a PMT (Hamamatsu H7421-40). The fourth channel was recorded simultaneously with the third channel to assess the morphology of the cells. All samples in this study were mounted on a motorized XY-axis piezo sample stage (SLC-24150-LC, SmarAct, Germany), equipped with a heating stage to maintain optimal conditions of temperature for cell samples during imaging.

The laser beam with ∼8 mW average power at the sample was scanned across a field-of-view (FOV) of 90 × 90 μm^2^ (spread across 512 × 512 pixels) for ∼2.5 s using 2 galvo mirrors, and the fluorescence signals at the two channels from different pixel locations were recorded simultaneously using a custom written LabVIEW (National Instruments) software. Each FOV was scanned only once to avoid photobleaching of the dye. Since the cells were in suspension, ∼5 min was allocated for the cells to settle down to the bottom of the imaging dish before imaging. Subsequently, fluorescence images were measured from 16 FOVs over ∼2 min time interval during each imaging session.

### CARS imaging

The transmission microscope for the detection of simultaneous CARS and multiphoton fluorescence signals was built on a commercial upright BX51 Olympus microscope (Olympus, Tokyo, Japan). The same dual output femtosecond laser source (as described before) was used to generate the laser excitation pulses [29,30]. From the tunable laser beam (660–1300 nm), the pump beam was centered at 800 nm, and the fixed wavelength 1040 nm beam served as the Stokes beam for CARS microscopy to excite the C-H vibrations at 2884 cm^−1^ (650 nm). The simultaneous absorption of 800 and 1040 nm photons contributed to the sum frequency excitation of AF488 dye at 452 nm.

The pump and the Stokes beam were overlapped spatially and temporally using a dichroic beamsplitter (Di02-980; Semrock) and a motorized translational stage (X-LSM050A-KX13A; Zabor Technologies, Inc.). A pair of 2D galvo-mirrors was used to raster scan the laser beams at the cell samples through a 40X/0.8 NA water immersion objective lens (LUMPLFLN; Olympus). The CARS signal was collected in the transmission geometry with a PMT (H7422-40; Hamamatsu) and a narrow bandpass filter centered at 650 nm (FF01-650/13/25; Semrock) to reject the excitation pulses. The power of each of the Stokes and pump beam at the sample was set to ∼10 mW.

Since the laser beam combination was able to excite the AF488 dye, we collected the 2PF emission signal in the epi direction with another PMT (H7422-40, Hamamatsu) and a set of dichroic mirrors and filters (FF01-571/72/25) to collect the fluorescence signal around 570 nm. Custom written LabVIEW (National Instruments) software was designed to scan the laser beams and acquire the CARS and fluorescence images simultaneously.

The galvo mirrors were set to a step voltage of 0.003 V to cover a FOV of 75 × 75 μm^2^ (400 × 400 pixels) using a pixel dwell time of 10 μs, which corresponds to 1.6 s per image. To enhance the signal, two preamplifiers, with a gain setting at 550 (PMT-4V3; Advanced Research Instruments Corp.), and a current-voltage converter were used to preamplify the CARS and fluorescence signals before they were acquired by the data acquisition system (PCIe-6351; National Instruments). A total of 20 frames for each FOV was collected and analyzed with MATLAB software (MathWorks).

### Image analysis

A single-cell analysis pipeline was developed for the drug uptake kinetics and its subsequent distribution. The analysis pipeline consisted of four main steps: (a) image preprocessing, (b) image segmentation, (c) feature extraction, and (d) downstream analysis. The images from both the inverted and transmission microscope were used in this analysis.

The 665 nm channel from the inverted microscope, which shows the cellular morphology from FAD and leaked AF488 fluorescence, was used for segmenting cell and nuclear regions. An identical segmentation process was used for the CARS images, which exhibited clear contrast between the cytoplasm and the nucleus. The channels that acquired the AF488-derived signals from both the inverted (554 nm) and the transmission (570 nm) microscopes were used to evaluate the drug uptake and its subsequent trafficking. The computer used for the analysis was a GPU workstation from Lambda, with an Intel Xeon W-2195 CPU, four Nvidia RTX 8000 GPUs with NVLink, and 256 GB of RAM. The operating system was Ubuntu 18.04, and Python and Jupyter Notebook platforms were used to build the analysis pipeline.

The first step in the analysis pipeline is image preprocessing. For image preprocessing, we trained an unsupervised deep learning model, Noise2Void [33], to denoise the 665 nm wavelength images. This model can effectively remove noise from 2PF images without removing intracellular details that are needed to segment out the nucleus. To further improve the contrast of cells and nuclei, we applied a Contrast Limited Adaptive Histogram Equalization (CLAHE) [34] process to the denoised images. The next step of image segmentation was split into two parts: cell segmentation and nucleus segmentation. We adopted the machine learning-assisted software tool, ilastik [35], for both segmentation tasks. The pixel classification module was used to label and segment cells from the images.

Cells that were cut off at the image edges were excluded from further analysis using the object classification module in ilastik. Based on the single cell images, the nucleus was segmented using a similar approach as cell segmentation. With the aim to quantitatively measure the ASO distribution within single cells, a radial mask was generated for the region between the cell edge and the nucleus edge. For each pixel in this region, we first interpolated a straight line that connects the pixel with the cell and the nucleus edge, like radius of a circle. Next, we calculated the distance of this pixel to the cell edge and the entire distance of the interpolated line that connects the pixel from nucleus edge to the cell edge. This implies that if the ratio is closer to one, the pixel is closer to the nucleus edge. Depending on the value of this ratio for each pixel, we divided each cell into five regions.

In the next step of feature extraction, the average cell fluorescence intensity Ī was calculated based on the 554 nm wavelength images. To compare the relative ASO uptake between different cell types, we extracted the fluorescence intensity for each cell as the first feature. We calculated the cell fluorescence intensity by excluding the fluorescence contribution from nucleus region from the entire cell. Next, we calculated the area of the cytoplasmic region by subtracting the area of the nucleus from the entire cell. By dividing the subtracted fluorescence intensity by the area of the cytoplasm, we obtained the normalized fluorescence intensity per cell.

This is shown in Equation 2, where $I_{c}$ and *I_n_* represent the fluorescence intensity of the whole cell and nucleus, respectively, and *S_c_* and *S_n_* represent the area of the whole cell and nucleus, and Ī being the normalized fluorescence intensity of the cell,

(2) $$Ī=\frac{I_{c}-I_{n}}{S_{c}-S_{n}}.$$

To understand the uptake kinetics of the ASOs in the cell cytoplasm, our next feature of interest is the area-normalized fluorescence intensities from individual regions obtained from the segmentation step. The average fluorescence intensity was calculated for each cell region ${Ī}_{R_{i}}$ using Equation 3, with $I_{R_{i}}$ and $S_{R_{i}}$ representing the total fluorescence intensity and the size, respectively, of the cell region *Ri*, resulting in

(3) $${Ī}_{R_{i}}=\frac{I_{R_{i}}}{S_{R_{i}}}.$$

We also calculated the percentage of drug in each cell region $Perc_{R_{i}}$ using Equation 4:

(4) $$Perc_{R_{i}}=\frac{I_{R_{i}}}{\sum^{I_{R_{j}}}}\times 100\%.$$

## Results

### Dose dependence of GalNAc-PS-ASO uptake

**FIG. 2.**
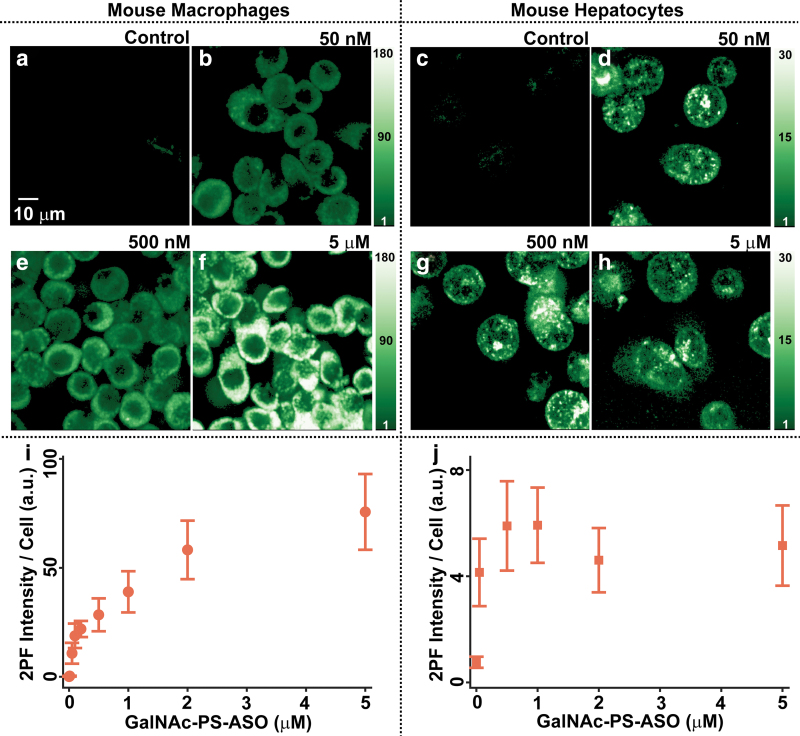
Concentration dependence of the AF488-conjugated GalNAc-PS-ASO cellular uptake. **(a, b, e, f)** Fluorescence intensity images of mouse macrophages (J774A.1 cells) reverse transfected with 0, 50 nM, 500 nM, and 5 μM GalNAc-PS-ASOs, respectively. **(c, d, g, h)** Fluorescence intensity images of PMHs reverse transfected with 0, 50 nM, 500 nM, and 5 μM ASOs, respectively. **(i, j)** Relative change in the average fluorescence intensity per cell with increasing GalNAc-PS-ASO doses in J774A.1 and PMH cells, respectively. Color images are available online.

The changes in the fluorescence intensity of J774A.1 mouse monocyte/macrophages and primary mouse hepatocytes (PMHs) as a function of the varying GalNAc-PS-ASO concentration are shown in Fig. 2. The J774A.1 cells that were reverse transfected with increasing doses of GalNAc-PS-ASOs were measured 24 h after the treatment. Figure 2a, b, e, and f shows the fluorescence images of J774A.1 cells exposed to 0 nM (Control), 50 nM, 500 nM, and 5 μM GalNAc-PS-ASO, respectively.

The PMHs that were reverse transfected with identical doses of GalNAc-PS-ASOs were measured 3 h after the treatment (Fig. 2c, d, g, h). This was done to reduce the effect of passive diffusion of ASOs inside the nonplateable cells as they suffer a natural loss in viability within 2–3 h post-thawing. In addition to this we also found an increasing number of apoptotic cells with a compromised cell membrane integrity at later times.

While murine macrophage-like cells showed a distinct increase in the fluorescence intensity with increasing ASO doses, PMHs displayed an initial rise in the fluorescence intensity up to 100 nM of GalNAc-PS-ASO. Beyond this dose, the intensity remained steady and comparable between the lower-end (500 nM) and the higher-end (5 μM) doses as shown in Fig. 2g and h.

To account for the concentration dependence quantitatively, individual cells were first identified in each image. The fluorescence intensities from all the pixels within those cells were integrated, and this sum was then divided by the area of the cells (total number of pixels occupied by the cells). Due to the size difference between the two types of cells, the number of J774A.1 cells analyzed (∼45 cells) was almost twofold higher for each data point than the PMHs (∼25 cells). Figure 2i and j shows the mean 2PF intensities from individual J774A.1 and PMH cells, respectively. The error bars show the standard deviation of cellular fluorescence intensity distribution.

An observation of a steady but nonlinear increase in the integrated fluorescence intensity as a function of GalNAc-PS-ASO concentration in J774A.1 cells (Fig. 2i) was expected, as the macrophages are known to engulf PS-ASOs and their GalNAc conjugated counterparts using scavenger receptors, [36,37], or by phagocytosis, regardless of their concentration [38]. This cell line has phenotypic characteristics similar to murine peritoneal macrophages [18,39], although the primary macrophages may show a faster and more intense response to certain pathogens [40]. Moreover, compared to PMHs, the J774A.1 cells may have a different number and recycling rate of membrane receptors that can recognize the GalNAc label.

The observation that PMHs showed no significant dose dependence starting with 100 nM of treatment (Fig. 2j) points to the hypothesis that there exists a threshold saturation concentration for GalNAc-PS-ASOs beyond which the uptake rate does not change. Supplementary Fig. S2 compares the uptake of 100 nm ASOs (PS-ASO and GalNAc-PS-ASOs in mouse hepatocyes counterstained with Hoechst).

GalNAc binding to ASGR occurs at the surface of the hepatocyte. Since each cell contains from 0.5 to 1.8 million receptors [6,41], of which about 5%–10% are present at the cell surface at any one time due to a rapid receptor turnover rate (∼15 min) [10,42], this represents an efficient endocytosis-mediated approach of GalNAc-conjugated ASO delivery to this vital organ [11,43,44]. Perhaps a saturating amount of extracellular GalNAc ligand favors internalized ASGR degradation (through late endosomes and lysosomes) versus recycling back to the plasma membrane. For instance, endocytic trafficking studies of epidermal growth factor receptor (EGFR) showed that clathrin-mediated endocytosis of EGFR has limited capacity and is saturated by the excess of EGF: EGFR complexes at the cell surface when high nonphysiological EGF concentrations are used [45].

### Kinetics of ASO uptake

Since there was no significant change in the intracellular uptake of GalNAc-PS-ASO in the primary hepatocytes beyond 100 nM (Fig. 2j), for further time-dependent ASO uptake studies we used a smaller nonsaturating ASO concentration (50 nM). The ASO uptake was measured immediately after the reverse transfection. As described under the Methods section, images were collected from three separate imaging dishes containing either 50 nM ASO or 50 nM GalNAc-PS-ASO-treated cells (either PMHs or J774A.1 macrophages). A fourth dish contained control cells that were reverse transfected with PBS buffer alone. The control sample measurements were conducted in between ASO and GalNAc-PS-ASO samples to ensure that the changes in the fluorescence intensity arose from the ASO uptake inside the cells.

**FIG. 3.**
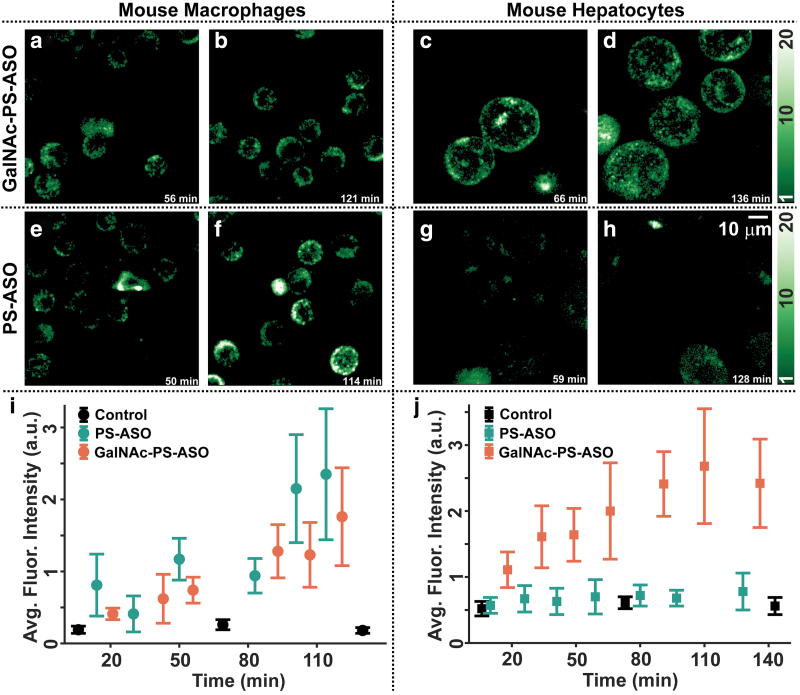
Comparison of the regular PS-ASO and GalNAc-PS-ASO uptake kinetics in mouse macrophages (J774A.1) and PMHs. **(a, b)** Fluorescence intensity images of GalNAc-PS-ASO-treated macrophages at 56- and 121- min postreverse transfection, respectively. **(c, d)** Fluorescence intensity images of GalNAc-PS-ASO-treated PMHs at 66- and 136-min postreverse transfection, respectively. **(e, f)** Fluorescence intensity images of ASO-treated macrophages at 50- and 114- min postreverse transfection, respectively. **(g, h)** Fluorescence intensity images of PS-ASO-treated PMHs at 59- and 128- min postreverse transfection, respectively. **(i)** and **(j)** show the change in the average fluorescence intensity per cell as a function of time after cell treatment with PS-ASOs (*teal symbols*), GalNAc-PS-ASOs (*orange symbols*), or PBS-alone (*black symbols*). Color images are available online.

The comparison of ASO uptake kinetics is shown in Fig. 3. Mouse J774A.1 macrophage images are presented in the top left panel (Fig. 3a, b, e, f), and the PMH cell images are in the top right panel (Fig. 3c, d, g, h). The first row (Fig. 3a–d) shows the fluorescence images of cells at different time points during the GalNAc-PS-ASO uptake process, whereas the second row shows the images of cells at different time points during the nontargeted (GalNAc-free) ASO uptake process.

Note that the earlier time point images for the ASO and GalNAc-PS-ASO uptake in J774A.1 cells are visually similar. However, at later time points the ASO-treated macrophages displayed a higher fluorescence intensity than the GalNAc-PS-ASO-treated counterparts. The scenario was completely different for the PMHs. Upon GalNAc-PS-ASO treatment conditions, these cells displayed intense fluorescence patterns at ∼1 h time point (Fig. 3c) post-transfection, suggesting a high rate of uptake. This intense fluorescence pattern persisted till the end of the measurement (Fig. 3d) (∼140 min). In contrast, the PS-ASOs showed weak fluorescence except for some regions in the less viable cells (Fig. 3h).

To compare the ASO uptake in both cell types, we assessed cell viability through visual inspection, then integrated the photon counts for each of these cells, and plotted the mean of this intensity distribution with time in Fig. 3i and j, following the same procedure as shown in Fig. 2i and j. The error bars correspond to the standard deviation of the integrated fluorescence counts from individual cells. Figure 3i shows the plots of ASO, GalNAc-PS-ASO, and PBS uptake in mouse J774A.1 cells. While the initial uptake rate was similar for ASO and GalNAc-PS-ASO, the ASO uptake rate increased at later time points, resulting in a more efficient ASO internalization inside these cells.

In contrast the GalNAc-PS-ASO uptake was significantly different from the PS-ASOs in the PMHs; as early as 20 min from the initiation of oligonucleotide delivery, the average fluorescence intensity increased drastically compared to the PS-ASO with time. The internalization of PS-ASOs was almost negligible as it hardly surpassed the background fluorescence intensity signals obtained from the control condition. These data suggest that due to the differences in ASGR quantity and recycling efficacy, PMHs are inclined to uptake GalNAc-PS-ASOs in much larger amounts over PS-ASOs, whereas the macrophages used in this study have a slight preference for the internalization of regular (GalNAc-free) ASOs.

### ASO uptake in primary rat hepatocytes

**FIG. 4.**
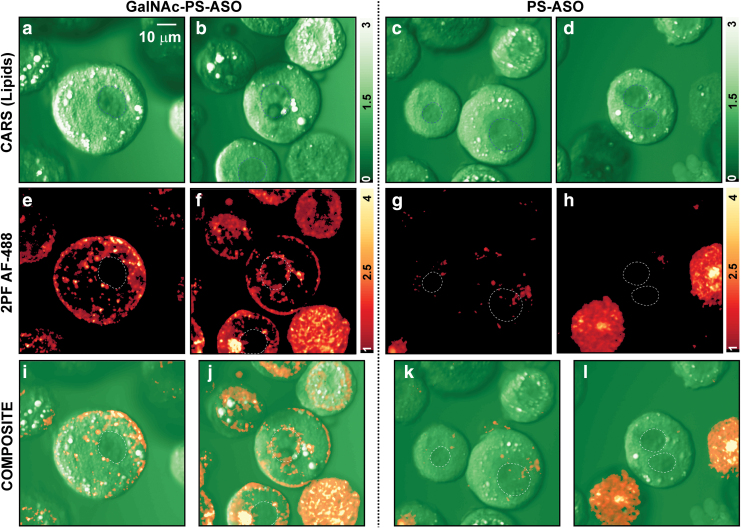
Differential uptake of GalNAc-PS-ASO and PS-ASOs in PRHs. **(a–d)** Broadband CARS images of PRHs after 2 h post-treatment with 50 nM GalNAc-PS-ASOs or 50 nM PS-ASOs. **(e–h)** Corresponding 2PF images that show the differential distribution of GalNAc-PS-ASOs with respect to regular PS-ASOs in PRHs. **(i–l)** Corresponding composite images of CARS (*green*) and 2PF (*red*). The nuclear regions of mononucleated or binucleated cells are highlighted with *dotted lines*. PRH, primary rat hepatocyte. Color images are available online.

To investigate if the differences between GalNAc-PS-ASO and ASO uptake translate in hepatocytes that are derived from other animal species, primary rat hepatocytes (PRHs) were reverse transfected with physiological 50 nM dose of PS-ASO and GalNAc-PS-ASOs. The visualized uptake of the GalNAc-PS-ASO-ASGR complexes is shown in the left panel of Fig. 4. Figure 4a–d shows representative CARS images of PRHs that received GalNAc-PS-ASOs (Fig. 4a, b) or PS-ASOs (Fig. 4c, d). The corresponding 2PF images are shown in Fig. 4e–h. Finally, the composite images overlaying the CARS (green) and the 2PF (red) images are shown in Fig. 4i–l.

The distribution of the GalNAc-PS-ASOs as seen from the 2PF images highlights a bright boundary, implying the formation of the GalNAc-ASGR complex on the plasma membrane surface and hence the beginning of the internalization of the ASOs into the cell. Like in PMHs, our data demonstrate a peripheral uptake of GalNAc-PS-ASOs in PRHs. The 2PF images also exhibit certain cellular morphologies that are filled with ASOs, irrespective of their label. These are dead/dying PRH cells, which permit a passive diffusion of any ASOs inside. These dead/dying cells were excluded from quantitative analysis.

The CARS images reveal the morphology of the cells in more detail. As the CARS excitation was set to the lipid -CH_2_ vibration at 2880 cm^−1^, it highlighted the lipid droplets as bright regions. In contrast, the nuclei of the cells exhibited a darker contrast and could be easily identified.

Mononuclear and binuclear hepatocytes are commonly observed in the liver. The binuclear cell population is ∼30%–40% in the normal rat liver [46]. Note that in some cell types, mononucleated cells are proliferative, whereas binucleated cells exit the cell cycle and no longer proliferate. The comparison of CARS and 2PF and the composite images revealed that aside from the cell membrane periphery, a significant number of GalNAc-PS-ASO molecules was found near the nuclear membrane. This internal distribution at 2 h after the treatment is indicative of the cellular trafficking mechanisms that happen postinternalization of GalNAc-bound ASGRs.

Previous studies have shown that the trafficking of the ASGR-ligand complex in the cell cytoplasm occurs through multistep endosomal and lysosomal (lipid) vesicles [47]. It is evident from the CARS-2PF composite images (Fig. 4i–l) that the larger lipid droplets do not play any role in either internalizing or trafficking of the GalNAc-PS-ASOs. This indicates the spontaneous formation of the lipid vesicles (lysosomes and endosomes) to facilitate endocytosis and further trafficking of the ASOs, rather than exploiting the lipid droplets as a resource.

In contrast to the GalNAc-PS-ASOs, the PS-ASOs showed a weak uptake by PRHs. After the elimination of dead cells from the 2PF image analysis, we found that only a select population of PRHs allowed the internalization of PS-ASOs. While it has been reported that the PS-ASOs are also uptaken through the ASGR mediated endocytosis [48], although less efficiently than the GalNAc-PS-ASOs, it is possible that the uptake is occurring in the hepatocytes that are at very early stages of apoptosis. However, it is well established that ASOs can be internalized into hepatocytes by other membrane receptors that have some ASO affinity, including clathrin-mediated, caveolin-mediated, and nonclathrin/noncaveolin mediated receptor uptake. The findings likely reflect these other less efficient membrane systems, which permit the uptake of limited amounts of ASOs at select regions of the cell.

### ASO trafficking kinetics using the individual cell analysis pipeline

**FIG. 5.**
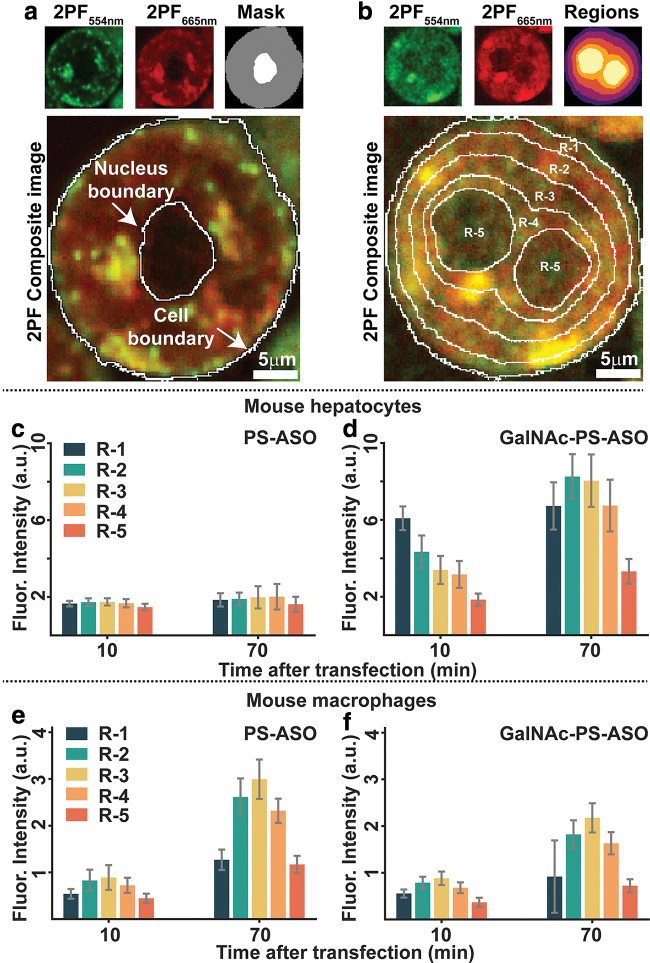
Cell region segmentation in the 2PF images and the average fluorescence intensity in each cell region at 10 and 70 min after ASO treatment. **(a)** Cell and nucleus segmentation results. **(b)** Radial mask generated based on cell and nucleus masks. Regions 1–5 in **(b)** represent five different cytoplasmic regions based on their distance from the nucleus/nuclei. **(c)** PS-ASO and **(d)** GalNAc-PS-ASO distribution within the subcellular regions of PMHs. **(e)** PS-ASO and **(f)** GalNAc-PS-ASO distribution within the subcellular regions of mouse macrophage J774A.1 cells. Color images are available online.

Following the image analysis pipeline outlined in the Methods section, we preprocessed the cell images, segmented both cells and their nuclei, and extracted features like the normalized fluorescence intensities for each cell Ī and their regional respective fluorescence percentage contributions $Perc_{R_{i}}$. The total number of segmented cells for PMHs and Mouse macrophage J774A.1 that were used in this analysis are shown in the Supplementary Figs. S3 and S4, respectively. Figure 5a shows a representative cell segmentation outcome following analysis from the two 2PF channels. The mask of a cell is shown in gray that also contains the nucleus mask (shown in white).

In the composite image both the nucleus edge and the cellular edges are identified. Figure 5b shows an instance of both the cell/nucleus segmentation with regional masking for a double nucleated cell. The regions are numbered from R-1 to R-5 with R-1 being the cell membrane region and R-5 being the nucleus. Figure 5c–f shows the features extracted from the five different regions from individual cells subject to different treatment conditions. Figure 5c and d shows the normalized fluorescence intensities from five different regions of PMHs reverse transfected with nontargeted and GalNAc-PS-ASOs, respectively, at times 10 and 70 min postreverse transfection. Figure 5e and f shows the same for J774A.1 cells.

Based on these feature values, we compared the amount of ASO uptake and the intracellular distribution of these ASOs at different time points in PMHs and J774A.1 macrophage cells. Like the abovementioned observation, this image analysis also revealed that PMHs internalize a significantly greater amount of GalNAc-PS-ASOs than PS-ASOs at both 10 min (*P* < 0.0001) and 70 min (*P* < 0.0001) after reverse transfection (Fig. 5c, d). As for macrophage cultures (Fig. 5e, f), not only were the uptake rates of ASOs and GalNAc-PS-ASOs similar, but the ASO distribution inside the cells within the first 10 min of treatment also followed a similar pattern (*P* = 0.39). However, at 70 min after treatment, the amount of intracellular ASO was significantly higher than the amount of GalNAc-PS-ASOs (*P* < 0.005), confirming the results of our imaging data.

**FIG. 6.**
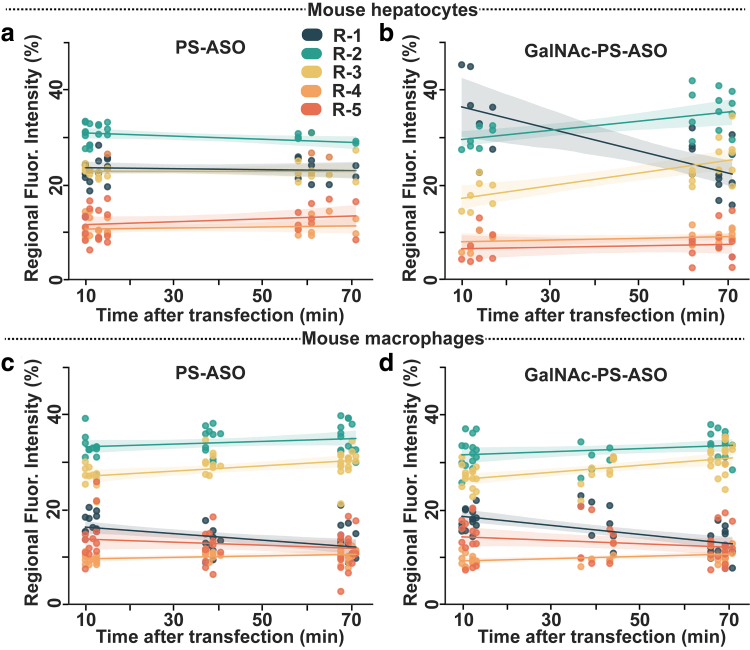
ASO uptake kinetics in cells. The percentage of PS-ASO **(a, c)** and GalNAc-PS-ASO **(b, d)** molecules in different cell regions at different times after treatment to PMHs **(a, b)** or mouse J774A.1 macrophages **(c, d)**. R1 represents the cell membrane region, and R5 represents the perinuclear region. R2–R4 represents the intermediate cytoplasmic regions. Color images are available online.

To have a better understanding of the ASO distribution within different cellular regions, we sought to analyze the fraction of the total amount of ASOs present in a specific subcellular region, rather than the total intensity from that region. Therefore, Equation 3 was used to generate Fig. 6, where each point represents the fraction of the total intensity for the cell membrane (R-1), outer cytoplasm (R-2), inner cytoplasm (R-3), perinuclear region (R-4), or nuclear region (R-5) of the cell. Figure 6a shows the time-dependent ASO distribution in PMHs. As observed in Fig. 5c, the ASO distribution in various cellular regions remained almost unchanged between 10 and 70 min post-treatment.

In the case of PMHs reverse transfected with GalNAc-PS-ASOs, the fluorescence intensity distribution (Fig. 5b) between the regions changes drastically between 10 and 70 min post-transfection. This triggers a sharp drop in the percentage of fluorescence intensity from R-1(cell membrane region) as seen in Fig. 6b suggesting that with the passage of time, an increasing number of GalNAc-PS-ASO molecules are transported to the inner regions of the cells. Once again, in the mouse J774A.1 macrophages (Fig. 6c, d), both the regular GalNAc-free ASOs and the GalNAc-PS-ASOs exhibited similar distribution patterns: (a) a decrease in the ASO concentration in the membrane region, (b) an increase in the cytoplasmic region, and (c) no increase/decrease in the perinuclear and nuclear regions of the cells.

## Discussion

The primary goal of this study was to track PS-ASO and GalNAc-PS-ASO uptake using two cell cultures as the first step to understand the observations from the clinical studies [16]. The multimodal imaging setup of CARS and 2PF modalities in conjunction with the image analysis pipeline made it uniquely possible to address these challenges. In this study, we have used single frequency CARS imaging coupled with fluorescence imaging to track and compare the uptake patterns of PS-ASO and GalNAc-PS-ASO in different populations of liver cells. We report here the dose- and time-dependent uptake, internalization, and regional distribution differences of GalNAc-PS-ASOs compared to PS-ASOs in immortalized mouse monocyte/macrophage J774A.1 cells and primary rodent (mice or rat) hepatocytes.

While the fluorescence images provided the information about the internalization and successive internalization of the PS-ASOs (with and without GalNAc) within the cells, the CARS images highlighted the cellular and nuclear morphology and the lipid distribution within them. Using both single cell analysis pipeline and average cellular metrics, derived from the multimodal optical images, we obtained a robust kinetics model for the internalization and the successive trafficking of the PS-ASOs. It is important to emphasize that the optical imaging is not sensitive to the 1% or less amount of ASOs that escape the endosomes during the intracellular trafficking process.

Our techniques measure the remaining 99% of ASOs which remain embedded in the travelling endosomes, until they are artificially disrupted due to the cellular manipulation. This reveals a shortcoming of the existing PS-ASO trafficking analysis in the cell cytoplasm: the biological insights into the endosomal/lysosomal contribution to the PS-ASO distribution are lacking even though we obtained the kinetics model.

The cellular uptake process of the PS-ASOs consists of mainly two steps: (a) adsorption on the cell surface (in this case the cell membranes) and (b) intracellular shuttling of the cargo. GalNAc-PS-ASOs bind to individual hepatocytes, which may have more than 5 × 10^5^ ASGR copies [6,8], with remarkable specificity. The ASGR with the GalNAc-PS-ASO ligand is shuttled rapidly into the cytoplasm through various endosomes and lysosomes, as evidenced by the rise in cytoplasmic concentration from Fig. 6b. The ASOs are subsequently driven more toward the perinuclear region containing the endoplasmic reticulum (Fig. 5d).

Macrophages, on the other hand, exhibit a completely different ASO uptake pattern, corresponding to a different ASO uptake mechanism. While macrophages may contain a few copies of transmembrane ASGR or its homologs [8], the adsorption mechanism is different from that observed in the PMHs and consistent with current dogma suggesting that ASO uptake in macrophages occurs through scavenger receptors.

Scavenger receptor mediated uptake of PS-ASOs in cells and tissues has been extensively studied and reported before [49,50] and it is the scavenger receptor SR-AI/II that plays an important role in internalizing the PS-ASOs for macrophages. In this study, we observe that the mouse J774A.1 macrophages exhibit similar initial uptake for both PS-ASO and the GalNAc-PS-ASO (Figs. 3 and 5) unlike the PMHs. In addition, the ASO uptake amount increases with an increase in ASO concentration (Fig. 2i).

The slow but continuous rate of endocytic ASO uptake can be attributed to weakly adhering PS-ASOs to the cell surface proteins or to the slow endocytic process of the PS-ASO protein complex into the cells. The effect of ASO uptake has been studied in monkey liver Kupffer cells previously [51–53]. These macrophages displayed a considerable number of vacuoles or granulated cytoplasm due to cytokine secretion.

While there was no evidence of the ASO presence in these tissue macrophage vacuoles [51], our macrophage data reveal bright granularities or vacuoles (Fig. 3f and Supplementary Fig. S5). This could either be from the fluorescent dye that got cleaved from the ASO due to the acidic environment or from the ASO deposits in these cells. This observation provides us with both challenges and opportunities to evaluate the ASO localization in animal tissues post-treatment using multimodal optical imaging.

While the GalNAc-PS-ASO uptake rate in J774A.1 cells was slower and continuous, the hepatocytes reached a rapid plateau with limited further GalNAc-PS-ASO intake. This indicates the presence of a saturating (∼100 nM) GalNAc-PS-ASO concentration for binding to the transmembrane ASGR for PMH. Since the ASGR expression varies significantly between PMH and fresh hepatocytes, we expect the saturation concentration of GalNAc-PS-ASO to vary proportionately, an important fact to be kept in mind when designing effective clinical dosing regimes for drugs acting through receptor-binding and clathrin-mediated receptor endocytosis pathways.

At early time points, both types of ASOs exhibited similar internalization kinetics in J774A.1 cells, but at later time points (after 100 min post-transfection), the intracellular content of PS-ASOs appeared slightly higher than the GalNAc-PS-ASOs, although both ASOs were excluded from the nuclear and perinuclear regions. The hepatocytes displayed a completely different trend. Immediately at the initiation of the treatment, GalNAc-PS-ASOs displayed an increased adherence to the cell membrane, indicating ASGR-mediated peripheral uptake, and achieved a steady state by 60–80 min.

In contrast, PS-ASOs adhered to the hepatocyte cell membrane sparsely in a focally intense pattern, causing the uptake rate to be slow and nearly comparable to the control (no treatment) conditions. Eventually these ASOs displayed random patches of adherence to the cell membrane. While maintaining high viability of the primary hepatocytes in suspension for 2–3 h remained challenging, the dead/dying cells showed classic signatures of passive ASO diffusion within the time frame of the measurement.

We developed an effective strategy to eliminate such cells from our analysis and report an overall fivefold increase in GalNAc-PS-ASO uptake in viable hepatocytes compared to the PS-ASOs. This value was similar to the one reported for the GalNAc-PS-ASO-treated mouse hepatocytes by Prakash, *et al.* [7]. While we have not exploited all the resources for addressing other biological key questions, we have been deliberate about keeping our goals strictly to identify the differences in ASO uptake between different types of liver cells in this study.

Further studies using freshly isolated primary hepatocytes, primary Kupffer cells, or liver stellate cells are needed to validate the robustness of differential ASO uptake effects in liver.

Although the biological functioning of the two systems, the *in vivo* animal model and *in vitro* cell culture, is entirely different, the specificity of the GalNAc ligand to target the ASGR was surprisingly similar, irrespective of the experimental system. Thus, we report the suitability of multimodal optical imaging to assess these simple cellular models to form the basis for further investigations with more complex cellular cocultures and with tissue and animal models.

## Data Availability

Data underlying the results presented in this article are not publicly available at this time but may be obtained from the authors upon reasonable request and through a collaboration agreement.

## Supplementary Material

Supplemental data

Supplemental data

Supplemental data

Supplemental data

Supplemental data
