# Supplementary material for: Differential Uptake of Antisense Oligonucleotides in Mouse Hepatocytes and Macrophages Revealed by Simultaneous Two-Photon Excited Fluorescence and Coherent Raman Imaging
Source: Nucleic Acid Ther. 2022 Jun 1;32(3):163–76. doi: 10.1089/nat.2021.0059 (PMC9221167; doi:10.1089/nat.2021.0059)

**Figure SI-1**. 1 mL of freshly thawed primary Sprague-Dawley male rat hepatocyte suspension (#RTCS10, TFS) was treated with 50 nM AF488-PS-ASO. After 15 min (A) or 5 h 15 min (B) of incubation in a complete media, the ReadyProbes™ Cell Viability Imaging Kit reagents nucleic acid dye Hoechst 33342 and propidium iodide (PI) were added as per manufacturer’s instructions. Cells were maintained in media for 15 additional minutes prior to spinning at 55×g for 2 min at RT. Cell pellet was resuspended in fresh dye-free media, overlaid onto the 35 mm Ibidi dish with #0 glass coverslip and the fluorescence images from DAPI (blue) and TexasRed (red) channels were co-registered using inverted Zeiss Axio Observer fluorescence microscope using auto-exposure settings at low 10X (upper panels) or medium 20x (bottom panels) magnifications. The estimated cell viability 0.5 h and 5.30 h post-reverse transfection was 85% and 25%, respectively.


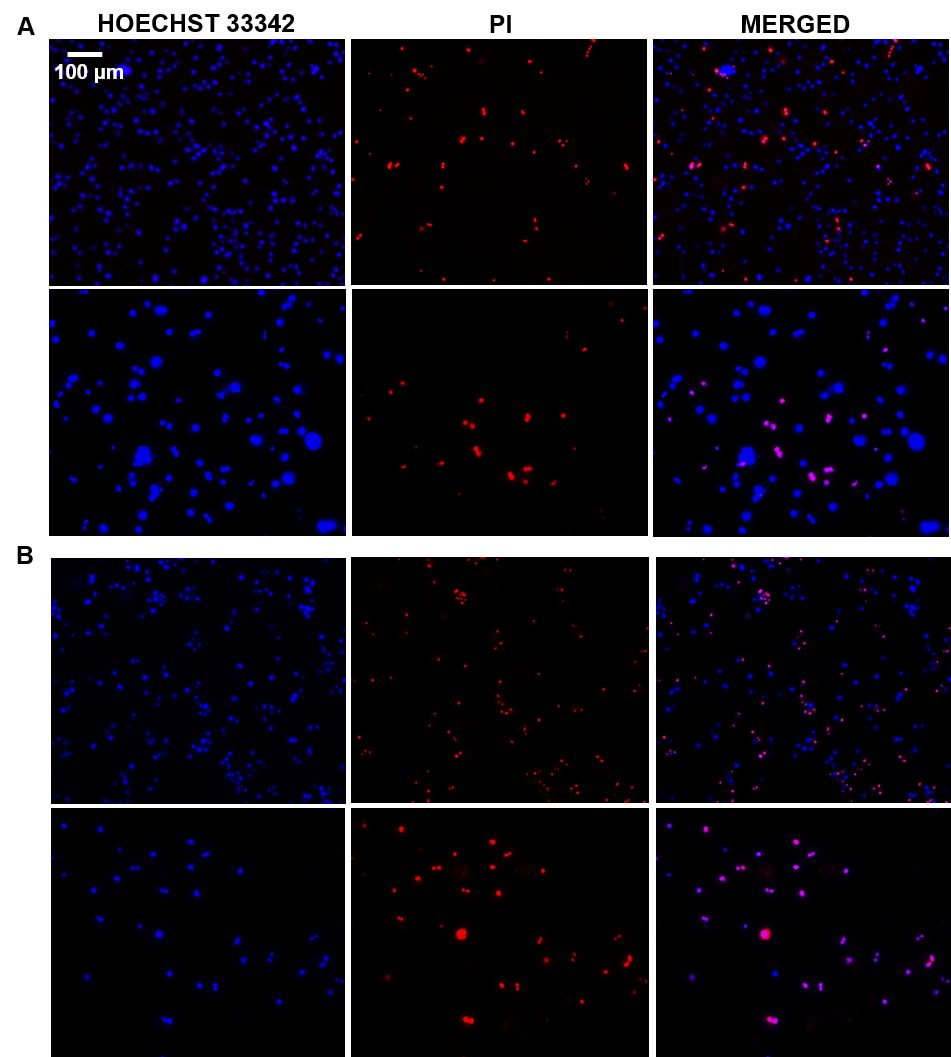

Supplement: Supplemental data [file Suppl_FigS1.docx]
