# Supplementary material for: Differential Uptake of Antisense Oligonucleotides in Mouse Hepatocytes and Macrophages Revealed by Simultaneous Two-Photon Excited Fluorescence and Coherent Raman Imaging
Source: Nucleic Acid Ther. 2022 Jun 1;32(3):163–76. doi: 10.1089/nat.2021.0059 (PMC9221167; doi:10.1089/nat.2021.0059)
Supplement: Supplemental data [file Suppl_FigS2.docx]

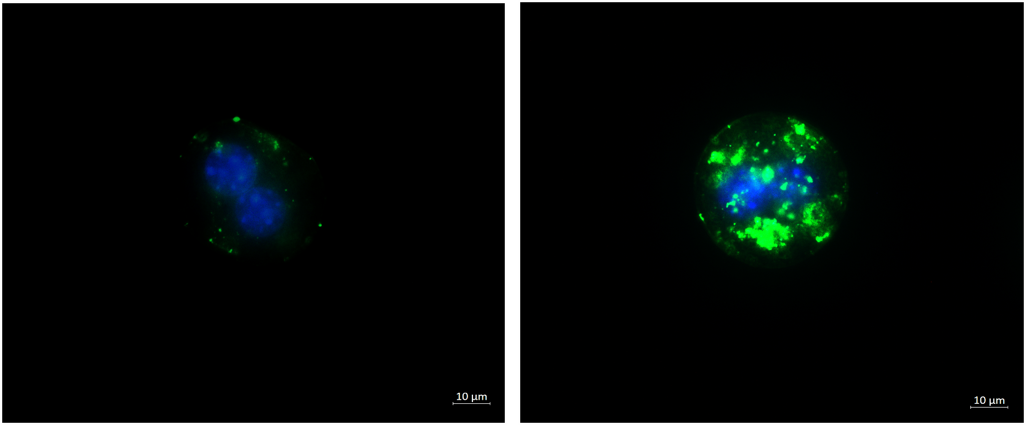
**Figure SI-2**. Representative high magnification (60x) fluorescence images of mouse hepatocytes that were fixed in 4% paraformaldehyde solution 2 h after the reverse transfection with 100 nM AF488-PS-ASO (left panel) and AF488-GalNAc-PS-ASO (right panel) (green channel) and counterstained with cell-permeant Hoechst 33342 ((2'-[4-ethoxyphenyl]-5-[4-methyl-1-piperazinyl]-2,5'-bi-1H-benzimidazole trihydrochloride trihydrate) nucleic acid stain (nucleus, blue channel). Scale bar = 10 µm.
