# Supplementary material for: Differential Uptake of Antisense Oligonucleotides in Mouse Hepatocytes and Macrophages Revealed by Simultaneous Two-Photon Excited Fluorescence and Coherent Raman Imaging
Source: Nucleic Acid Ther. 2022 Jun 1;32(3):163–76. doi: 10.1089/nat.2021.0059 (PMC9221167; doi:10.1089/nat.2021.0059)

**Figure SI-4**. Mouse macrophage J774A.1 cells under different treatment conditions segmented from 2PF-554nm and 2PF-665nm channels used in the single cell analysis.


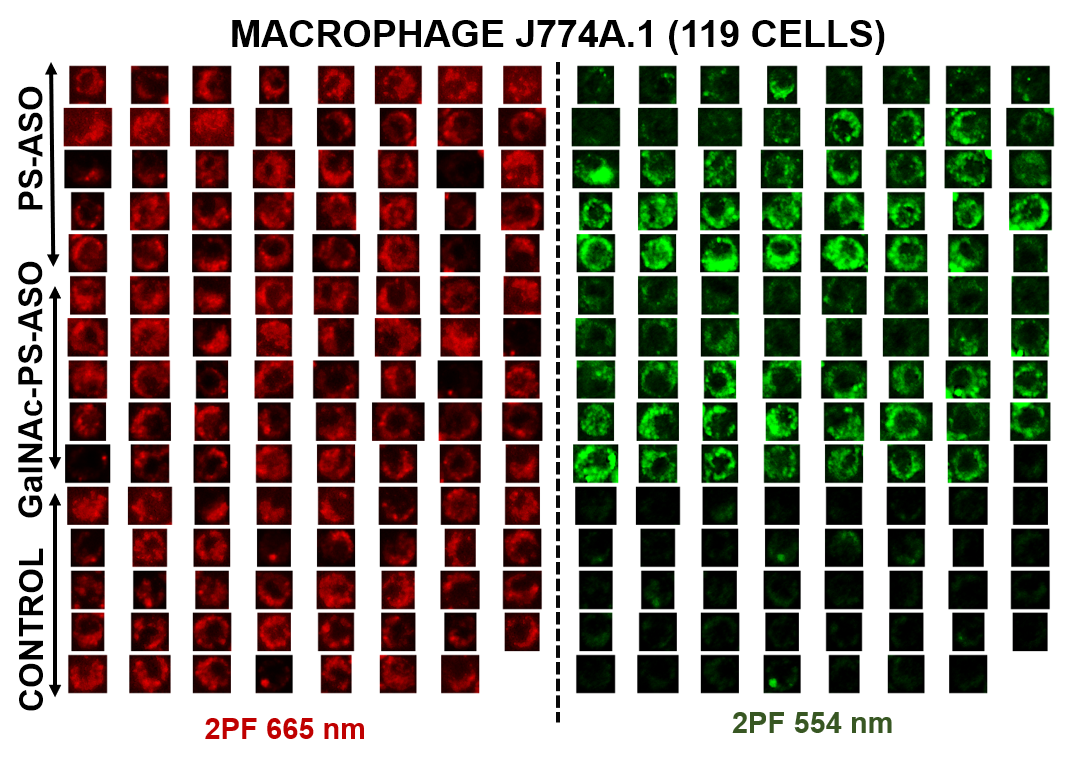

Supplement: Supplemental data [file Suppl_FigS4.docx]
