# Supplementary material for: Differential Uptake of Antisense Oligonucleotides in Mouse Hepatocytes and Macrophages Revealed by Simultaneous Two-Photon Excited Fluorescence and Coherent Raman Imaging
Source: Nucleic Acid Ther. 2022 Jun 1;32(3):163–76. doi: 10.1089/nat.2021.0059 (PMC9221167; doi:10.1089/nat.2021.0059)
Supplement: Supplemental data [file Suppl_FigS5.docx]

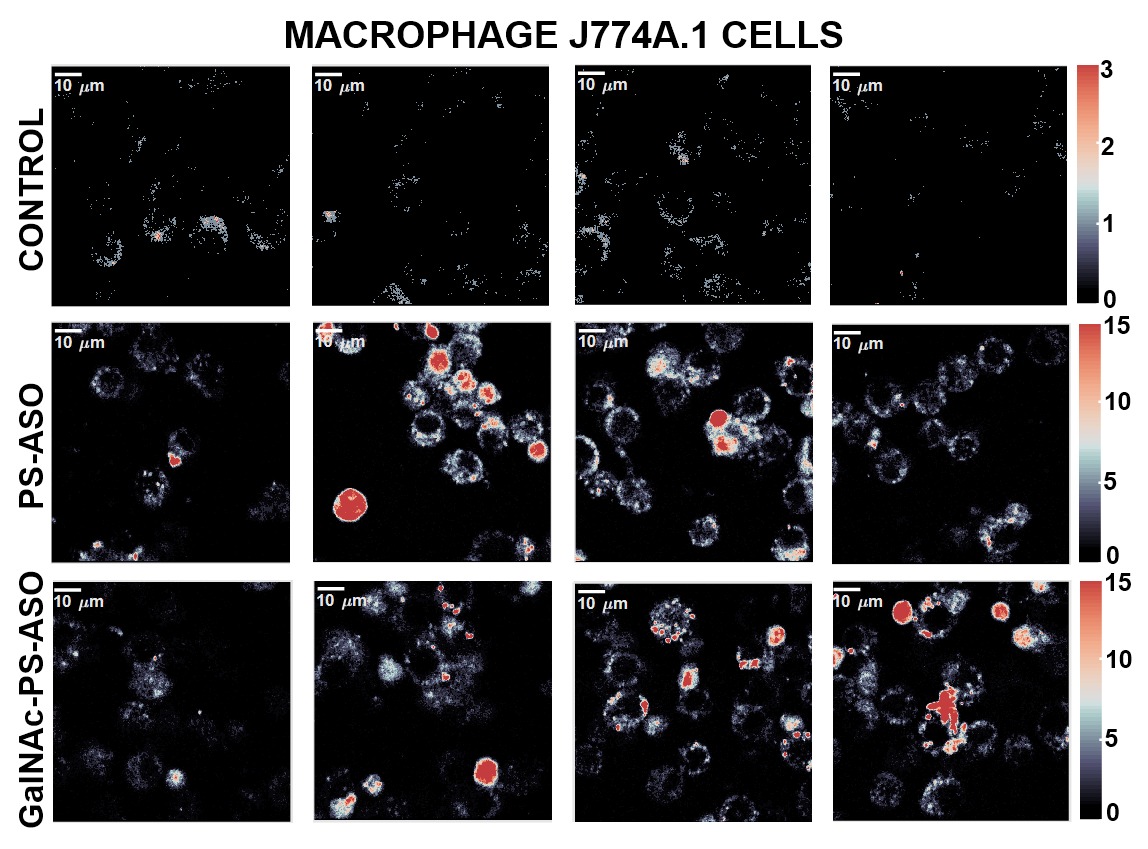
**Figure SI-5.** 2PF intensity images of mouse macrophage J774A.1 cells measured at 550 nm under different treatment conditions. Upon ASO/GN3-ASO uptake the macrophage cells exhibit a vacuolated granular morphology filled with ASOs.

**100 µm**
